# Matrix elasticity-modified scaffold loaded with SDF-1α improves the *in situ* regeneration of segmental bone defect in rabbit radius

**DOI:** 10.1038/s41598-017-01938-3

**Published:** 2017-05-10

**Authors:** Guobao Chen, Yonggang Lv

**Affiliations:** 1 Key Laboratory of Biorheological Science and Technology (Chongqing University), Ministry of Education, Bioengineering College, Chongqing University, Chongqing, 400044 P. R. China; 2 Mechanobiology and Regenerative Medicine Laboratory, Bioengineering College, Chongqing University, Chongqing, 400044 P. R. China

## Abstract

The effectiveness of stem-cell based therapy has been hampered by the limited availability of stem cell sources, immune rejection, and difficulties in clinical adoption and regulatory approval. These obstacles can be partially circumvented by using *in situ* tissue engineering that recruits the endogenous stem/progenitor cells and provides cues to direct stem cell phenotype. Here, decellularized bone scaffold is mechanically modified by coating of collagen (Col)/hydroxyapatite (HA) mixture with optimal ratio and loaded with chemokine stromal cell-derived factor-1α (SDF-1α), in which endogenous stem cell recruitment can be improved by chemokine and stem cell fate can be regulated by matrix elasticity of the scaffold. This study shows that mesenchymal stem cells (MSCs) osteogenesis *in vitro* was enhanced by matrix elasticity and SDF-1α, and endogenous MSCs recruitment in subcutaneous implantation of rat was increased by the release of SDF-1α from the scaffold, and bone regeneration in rabbit large bone defect model was significantly improved by matrix elasticity and SDF-1α. In short, this study provides a new insight for developing novel engineered cell-free bone substitutes by mechanical modification for tissue engineering and regenerative medicine.

## Introduction

Stem-cell therapy holds great promise in tissue engineering and regenerative medicine. Much attention has been focused on pluripotent stem cells and the use of their derivatives for therapeutic purposes. However, several uncertainties, such as tumorigenicity, immune rejection and high mortality after transplantation, hinder their clinical applications^1, 2^. An attractive alternative is to harness the potential of endogenous stem/progenitor cells for direct use in repair and regeneration^3^. In repair of bone defect, how to effectively utilize the endogenous stem cells has attracted more and more attention of various scholars^4–6^. Although the body possesses inherent mechanisms that guide stem cells to the injury region for regeneration by hypoxia and inflammatory response in early stage of bone fracture^7^, these endogenous processes are often insufficient to achieve full tissue repair because the recruiting stems cells are still seldom and recruitment mainly occurs in the early stage of bone fracture^8^. Therefore, development of *in situ* tissue engineering techniques needs to potentiate and prolong the body’s own repair capacity of substantial recruitment of autologous stem cells to the defect site and their tissue-specific differentiation.

Matrix elasticity has emerged as a key mechanical cue in osteogenic differentiation of stem cell *in vitro* ^9–13^ and can be a possible *in situ* approach to actively amplify intrinsic tissue repair processes by the optimal properties of matrix microenvironment. A recent study encapsulated human mesenchymal stem cells (MSCs) in three-dimensional (3D) void-forming hydrogel and implanted them into a rat xenograft cranial defect model^14^. This study suggests that mechanical cues can be harnessed to direct stem cell behaviors *in situ* for bone regeneration. However, it is hard to use hydrogel in large bone defects due to the poor mechanical strength of the hydrogel. For example, the size range of large bone defects in rabbit radius is 10–20 mm. Decellularized bone can avoid this mismatch and has natural porous microstructure and 3D interconnectivity, which are beneficial to migration and infiltration of endogenous stem cells. In our previous study^15^, novel 3D scaffolds with different matrix elastic modulus (6.74 ± 1.16 kPa, 8.82 ± 2.12 kPa, and 23.61 ± 8.06 kPa) but same microstructure have been successfully fabricated by coating decellularized bone with collagen (Col)/hydroxyapatite (HA) mixture in different collagen ratios. Our study has proved that the scaffold with optimal matrix elastic modulus (23.61 ± 8.06 kPa) can promote the osteogenic differentiation of MSCs *in vitro* and enhance the new bone formation *in vivo*. However, the number of recruiting stems cells was still low when the scaffold is lack of chemotaxis effect on endogenous stem cells in our previous study^15^. Fortunately, our *in vivo* study found that the bone graft areas had higher level of expression of stromal cell-derived factor-1α (SDF-1α) than the host bone areas during bone repair^16^. Thus, we suppose that SDF-1α may be one of the key players for MSCs recruitment in bone repair process.

Interaction between the chemokine SDF-1 (also known as CXCL12) and its receptor, CXCR4, is important in regulating stem cell recruitment and homing^17^, which has been reported to be participate in the regeneration of various tissues and organs such as heart^18, 19^, vascular^20^, tendon^21^, cartilage^22^, and so on. Furthermore, during the last decade, accumulating data have supported an emerging hypothesis that SDF-1/CXCR4 axis also plays a pivotal role in the recruitment of MSCs both *in vitro* and *in vivo* ^23–25^. Among these, Sordi *et al*.^26^ showed that MSCs expressing the CXCR4 receptor are strongly attracted by SDF-1 in a dose-dependent manner. Furthermore, SDF-1 has been demonstrated to participate in the activation of those signal transduction pathways in MSCs involved in the regulation of directional cell migration^27^. Iannone *et al*.^28^ also found that the concentration gradient feature and dose of SDF-1 signal effectively affect CXCR4 expression and production of human MSCs in a 3D collagen gel *in vitro*. Therefore, *in situ* bone regeneration can be partially circumvented by using the chemokine SDF-1α that can recruit endogenous stem cells and then osteogenic differentiation *in situ* can be achieved through a synergistic effect of appropriate mechanical cues.

We hypothesize that incorporation of SDF-1α in 3D scaffold with optimal matrix elasticity can recruit endogenous MSCs into the scaffold, induce the osteogenic differentiation of MSCs, and thus promote the bone regeneration *in situ*. In order to test this hypothesis, the present study will (1) fabricate and characterize a composite by loading 3D scaffold with optimal matrix elasticity with SDF-1α, (2) investigate the synergistic effects of SDF-1α and matrix mechanics on the fate of MSCs *in vitro*, (3) monitor and quantify the effects of SDF-1α on MSCs and inflammatory cells recruitment after subcutaneous transplantation in rats, and (4) evaluate the repair efficacy of the composite scaffold in rabbit large bone defect model *in vivo*.

## Materials and Methods

### Isolation and culture of rat MSCs

Primary rat bone marrow-derived MSCs were isolated from four-week-old Sprague-Dawley (SD) rats weighing 130 ± 10 g by flushing femurs and tibias and further cultured and identified following the method outline in our previous publication^29^. All animal work procedures were carried out according to the protocols approved by Chongqing University Animal Care and Use Committee. Cells were expanded in at 37 °C with 5% CO_2_ in low glucose Dulbecco’s Modified Eagle Medium (DMEM) (Gibco, USA) containing 10% fetal bovine serum (Gibco, USA) to the third passage, and used for the following experiments.

### Preparation of SDF-1α/Col/HA scaffold

Decellularized bone scaffold and Col/HA scaffold (Col 0.7/HA 22) were fabricated and characterized as reported previously^15^. Based on the Col/HA scaffold mentioned above, the SDF-1α/Col/HA scaffold was prepared and loaded with recombinant human SDF-1α (PeproTech, USA)^15, 21^. Briefly, SDF-1α solution at a concentration of 200 μg/mL^30, 31^ was uniformly mixed with collagen type I solution (0.7 wt%) at a ratio of 1:1000 (total volume of 7 mL) at 4 °C overnight in the dark. Then, defined collagen solution was mixed with a calculated amount of HA powder to prepare 22 wt% mixtures of HA in the collagen solutions under constant stirring by a magnetic stirring apparatus at 4 °C in the dark for 3 days. The prepared decellularized bone scaffold (diameter = 10 mm, thickness = 5 mm) was dipped in the mixture of SDF-1α/Col/HA mixture at 4 °C in the dark for overnight and shaken gently. After SDF-1α/Col/HA mixture was soaked evenly on the surface of scaffold, the scaffold was freeze-dried at −55 °C and 15 Pa by freeze dryer (Boyikang, China) for 24 h. Finally, the scaffolds were sterilized by ^60^Co γ irradiation (25 k Gay) and stored at −80 °C before further application.

### Characterization of SDF-1α/Col/HA scaffold

#### *In vitro* release of SDF-1α

To evaluate the release profile of SDF-1α from SDF-1α/Col/HA scaffold *in vitro*, enzyme-linked immunosorbent assay (ELISA) was performed according to the manufacturer’s protocol (NeoBioscience, China). Briefly, the SDF-1α/Col/HA scaffold was soaked into a 5 mL tube with 3 mL phosphate buffered saline (PBS) (n = 5). The tubes were protected from light and incubated at 37 °C in a continuous horizontal shaker. At predetermined time points (6 h, 12 h, 24 h, 2 days, 3 days, 5 days, 7 days, 10 days, 14 days, 21 days, 28 days, 35 days, 42 days, and 49 days), medium samples were taken from the tube and immediately frozen at −80 °C, and the same volume of fresh PBS was added to the tube to replace the removed aliquot. The SDF-1α content in the supernatant was assessed using an ELISA kit.

#### Immunofluorescence staining for SDF-1α

In order to verify whether the SDF-1α has been coated on the scaffold, immunofluorescent staining was performed by using SDF-1α antibody (Boster, China; diluted 1:50) and incubated overnight at 4 °C in a humidified chamber. The secondary antibody was applied with an Alexa Fluor 488-labled donkey anti-rabbit IgG (Molecular Probes, USA; diluted 1:500). Three samples for each group were imaged by the fluorescence microscope (Olympus IX71, Japan).

### Cell seeding on scaffold *in vitro*

Sterile scaffolds were seeded with 20 μL cell suspension containing 4 × 10^5^ cells. Scaffolds were flipped every 15 min for 1 h to achieve uniform cell distribution. Each time, 10 μL of culture medium was added to the surface of scaffold to prevent the cells from drying out. Cells were allowed to adhere after incubation for 1 h and then the culture medium was filled up to 6 mL in each well. Cell culture was maintained in an incubator at 37 °C, 5% CO_2_, and 95% humidity. The culture medium was changed every three days.

### Viability of cells seeded on SDF-1α/Col/HA scaffold

The cell viability on different scaffolds was assessed by live/dead assay. On the third day, the first week and the third week, scaffolds were harvested and incubated in 2 μM calcein AM and 4 μM ethidium homodimer-1 solutions for 30 min in the dark, as indicated by the manufacturer’s instructions (LIVE/DEAD Viability/Cytotoxicity Kit, Molecular Probes, USA). After washing 3 times with PBS, the fluorescence was visualized using a fluorescence microscope (Olympus IX71, Japan).

### Subcutaneous implantation

The cylindrical-shaped scaffolds (diameter = 5 mm, thickness = 4 mm) were prepared and sterilized by ^60^Co γ irradiation (25 k Gay) before implantation. SD rats (male, body weight 230 ± 5 g) were housed in wire bottomed cages in a temperature- and light-controlled room. The use of rats was approved by Third Military Medical University Animal Care and Use Committee. The rats were anaesthetized with 7% chloral hydrate (0.5 mL/100 g). A decellularized bone scaffold without coating, a Col/HA scaffold and a SDF-α/Col/HA scaffold were inserted into the subcutaneous pockets at the top, middle, and bottom on the dorsum of the rat, respectively (four animals for each time point). After 5 days, 10 days, 28 days and 56 days, rats were sacrificed, and the implanted scaffolds were removed naturally with the surrounding tissue.

### Rabbit radius defect repair

New Zealand white rabbits from both sexes weighing an average of 3 kg were used and randomly divided into four groups, (A) defect repair without any scaffold; (B) defect repair with a pure decellularized bone scaffold; (C) defect repair with a Col/HA scaffold; (D) defect repair with a SDF-1α/Col/HA scaffold (twelve animals for each time point). Animal experiments were carried out in the Animal Laboratory, Daping Hospital, Third Military Medical University, Chongqing, China. All animal experimental procedures were carried out according to the protocols approved by Third Military Medical University Animal Care and Use Committee. The surgery was carried out under sterile condition to establish a model with critical-sized segment defect (see Supplementary Fig. S1), started with ear vein anesthesia of rabbit by 2% pentobarbital sodium (30 mg/kg, Sigma, USA). After shaving off hair on the forelimb, a skin and musculature incision with 20 mm was made at the anteromedial aspect. Then, a complete bone defect of 15 mm in length, which included the periosteum, was made in the middle segment of the radius by using a hard drill. All bone debris and interosseous membrane at the defect site were washed and wiped away with physiological saline. The ulna was intact for mechanical stability. The cylinder scaffold (diameter = 5 mm, length = 15 mm) was inserted into the bone defect without any fixation device. After implantation, the wounds were carefully washed with physiological saline, and the incisions were sutured in full thickness. After 1 month and 2 months, the rabbits were sacrificed to obtain the bone tissue specimens in defective areas.

### Hematoxylin-eosin (H-E) staining and Masson trichrome staining

The samples were fixed in 4% paraformaldehyde at room temperature and then decalcified with 12% EDTA-2Na, dehydrated with graded ethanol, immersed in xylene, and embedded in paraffin. Paraffin was cut into 5 μm longitudinal sections and deparaffinized for H-E (Beyotime, China) and Masson trichrome staining (Nanjing Jiancheng Biotechnology Institute, China). Three samples for each group were observed with a light microscope (Olympus IX71, Japan).

### Immunohistochemistry for osteopontin (OPN) and osteocalcin (OC)

Sections were deparaffinized with xylenes and rehydrated in a descending series of ethanol concentrations. Citrate buffer (10 mM, pH 6.0) was used to do antigen retrieval at 95 °C for 25 min. After antigen retrieval, sections were allowed to cool down to room temperature. Sections were blocked for endogenous peroxidase activity with 0.3% hydrogen peroxide for 20 min. Nonspecific protein binding was blocked by incubation with 2% bovine serum albumin and then incubated with the anti-OPN (Abcam, USA; diluted 1:300) or anti-OC (Bioss, China; diluted 1:50) overnight at 4 °C in a humidified chamber. The secondary antibody was applied, carried horseradish peroxidase and developed according to the manufacturer’s protocol (IHC staining module, Beijing Zhongshan Biotechnology, China). Three samples for each group were analyzed by an inverted light microscopy (Olympus IX71, Japan).

### Immunofluorescence staining

After subcutaneous implantation for 5 days, 10 days, 28 days and 56 days, samples were fixed in 4% paraformaldehyde, and then decalcified in 12% EDTA-2Na. The sample was cut in half, and one for paraffin sections while the other for frozen sections. Frozen sections (8 μm thick) were then subjected to immunofluorescence for antibody of rabbit anti-rat CD29, CD34, CD44, CD68, CD90, and CD105 (Bioss, China; diluted 1:100). The Alexa Fluor 488-labled donkey anti-rabbit IgG (Molecular Probes, USA; diluted 1:500) was used as the second antibody. DAPI (1 μg/mL) staining was used to reveal the nuclei.

### X-ray analysis

The harvested radius-ulna specimens at 1 and 2 months were examined by X-ray machine (Faxitron MX-20, USA) to evaluate new bone formation in defective sites using Faxitron MX-20 specimen radiography system at the energy value of 30 kV for 90 s. At least three specimens in each group were X-rayed at each research time point.

### Micro-computed tomography (μ-CT) evaluation

The defective radius and adjoining ulna were harvested and scanned by a μ-CT imaging system (Scanco medical AG vivaCT40, Switzerland). 3D μ-CT images were reconstructed using the special software μ-CT v6.1 of Scanco medical AG. Slice increment is 19.5 μm. Meanwhile, in order to observe the repair effect directly on the middle of radial bone defects, the middle section images were selected from all the 2D section view images at 2 months with different implants.

### Statistical analysis

Each experiment was performed at least three times. All values were means ± SD (standard deviation). Comparisons between groups were made by one-way analysis of variance (ANOVA). A statistical significance was assigned *p < 0.05 and **p < 0.01, respectively.

## Results and Discussion

### Fabrication and SDF-1α immobilization validation of SDF-1α/Col/HA scaffold

**Figure 1:**
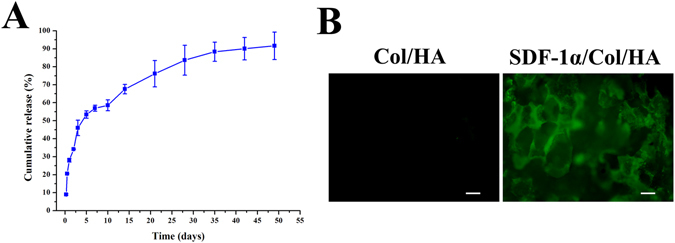
Time course of SDF-1α release (**A**) and immunofluorescence staining for SDF-1α immobilization (**B**) on the surface of a SDF-1α/Col/HA scaffold. The scale bar indicates 100 μm.

The stability and *in vitro* release of immobilized SDF-1α on SDF-1α/Col/HA scaffold were evaluated by ELISA. The cumulative release profile showed that the SDF-1α could be released over 49 days (Fig. 1A). A burst release of SDF-1α (20.53 ± 0.05%) was observed within the first 12 h, after which the release continued slowly, reaching about 53.42 ± 2.01% after 5 days. And from day 5 onward, the scaffold loaded with SDF-1α exhibited a sustained release profile, which resulted in 83.64 ± 8.27% cumulative release after 49 days (Fig. 1A). During this period, the SDF-1α/Col/HA scaffold showed no apparent visual signs of degradation. Immunofluorescence staining for SDF-1α in the SDF-1α/Col/HA scaffold showed that SDF-1α was evenly immobilized onto the Col/HA scaffold (Fig. 1B). The Col/HA scaffold without SDF-1α was also stained with SDF-1α antibody and showed negative staining (Fig. 1B).

### Cell compatibility of SDF-1α/Col/HA scaffold

**Figure 2:**
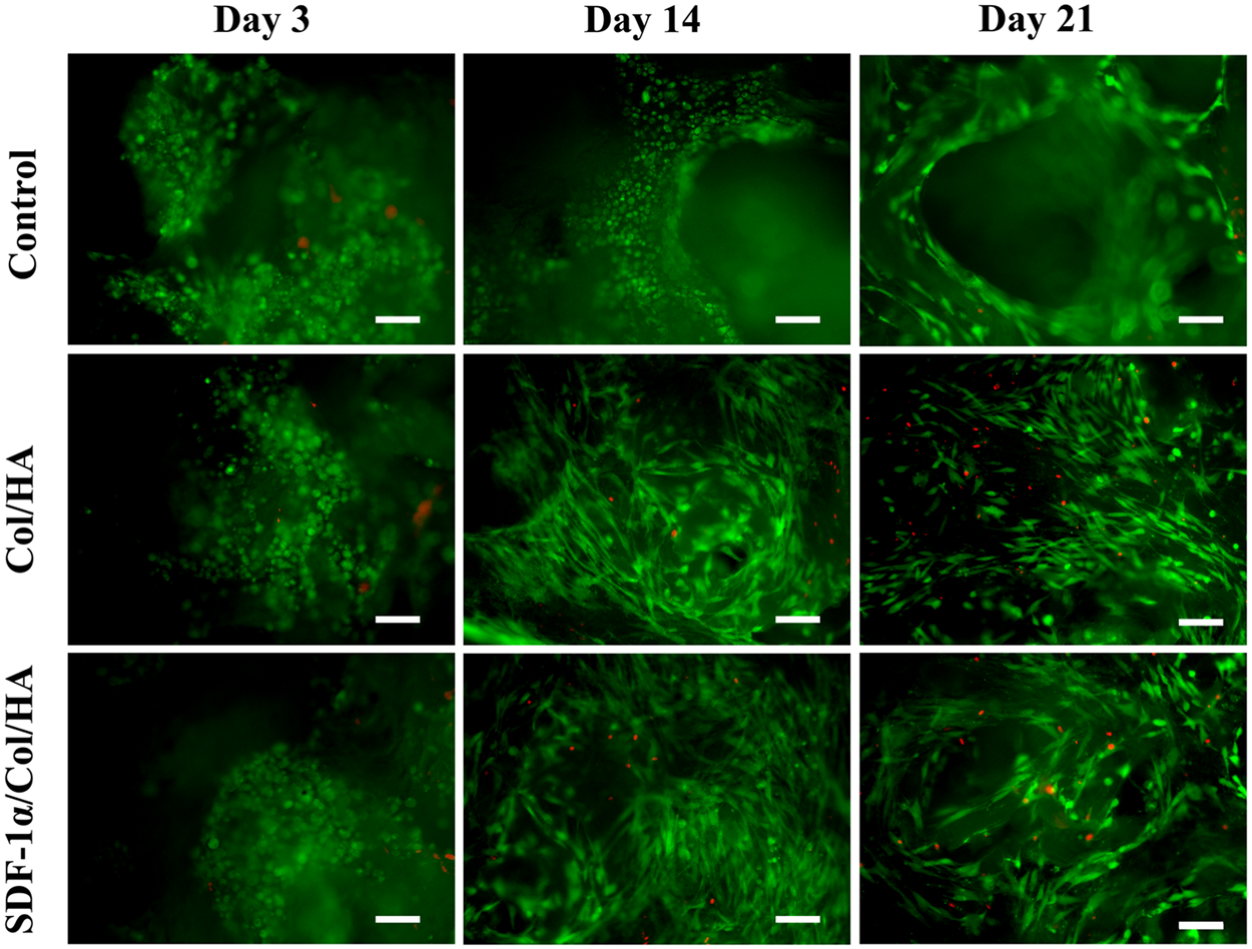
Live/dead assay of the scaffolds along the cultivation process. Green indicates live cells, and red indicates dead cells. The scale bar indicates 50 μm.

Live/dead assay was used to evaluate the MSCs viability by quantifying the live and dead cells on the scaffolds (Fig. 2). Live/dead assay revealed uniform distribution of MSCs throughout the scaffolds in periods of 3 days, 2 weeks, and 3 weeks. Most of the MSCs were viable after culture for 3 weeks with expansion medium. On the one hand, the slight increase in cell number on week 2 reflected cell proliferation. On the other hand, a slight increase of the dead cells on week 3 may be indicated cell density-dependent limitation of growth in 3D porous scaffold during prolonged culture *in vitro* ^32, 33^.

### Effect of SDF-1α/Col/HA scaffold on the osteogenic differentiation of MSCs *in vitro*

**Figure 3:**
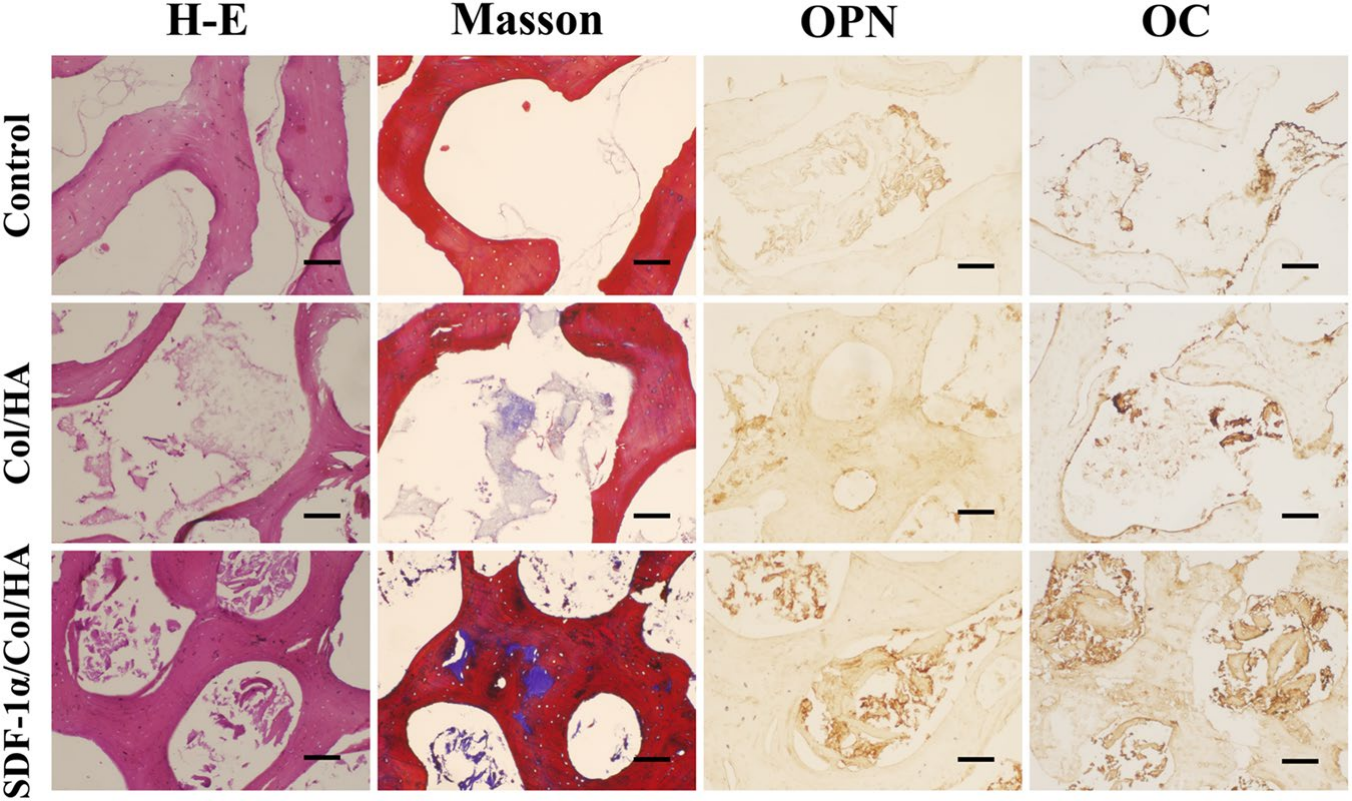
Histological evaluation of cultured constructs after culture for 3 weeks. Images from left column to right column are H-E staining, Masson trichrome staining, and immumohistochemical staining of OPN and OC. The scale bar indicates 50 μm.

To investigate the effect of SDF-1α/Col/HA scaffold on the osteogenic differentiation of MSCs, the expressions of two specific protein markers OPN and OC were examined. Meanwhile, the extracellular matrix (ECM) secretion and collagen deposition of MSCs on the scaffold were also evaluated by H-E staining and Masson trichrome staining. Interestingly, the positive immunohistochemical staining of OPN and OC in the SDF-1α/Col/HA scaffold were higher than those in other groups after culture for 3 weeks (Fig. 3). After culture for 3 weeks, the SDF-1α/Col/HA scaffold exhibited much more ECM deposition and collagen deposition than other groups (Fig. 3). These results suggested that the SDF-1α/Col/HA scaffold has a positive effect on osteogenic differentiation and ECM secretion of MSCs. Our previous study found that the Young’s modulus of the Col/HA scaffold is about 23.61 ± 8.06 kPa and this elastic modulus affects the MSC-scaffold interaction and then promotes the osteogenic differentiation of MSCs^15^. In addition, previous study reported that SDF-1 had a regulatory role in bone morphogenetic protein 2 (BMP 2)-induced osteogenic differentiation of MSCs, as blocking of the SDF-1 signaling inhibited the BMP 2-induced early expression of Runt-related factor-2 (Runx2) and osterix, two transcription factors required for differentiation of MSCs into osteoblasts^34^. The decellularized bone used in the present study is a nature derived material and has long been recognized as an osteoinductive scaffold due in large part to its BMP 2 content^35, 36^. The present results confirmed that SDF-1 involved in the osteogenic differentiation of MSCs *in vitro*.

### Host response of SDF-1α/Col/HA scaffold to subcutaneous implantation

#### Utilization of stem cell makers to identify MSCs *in vivo*

**Figure 4:**
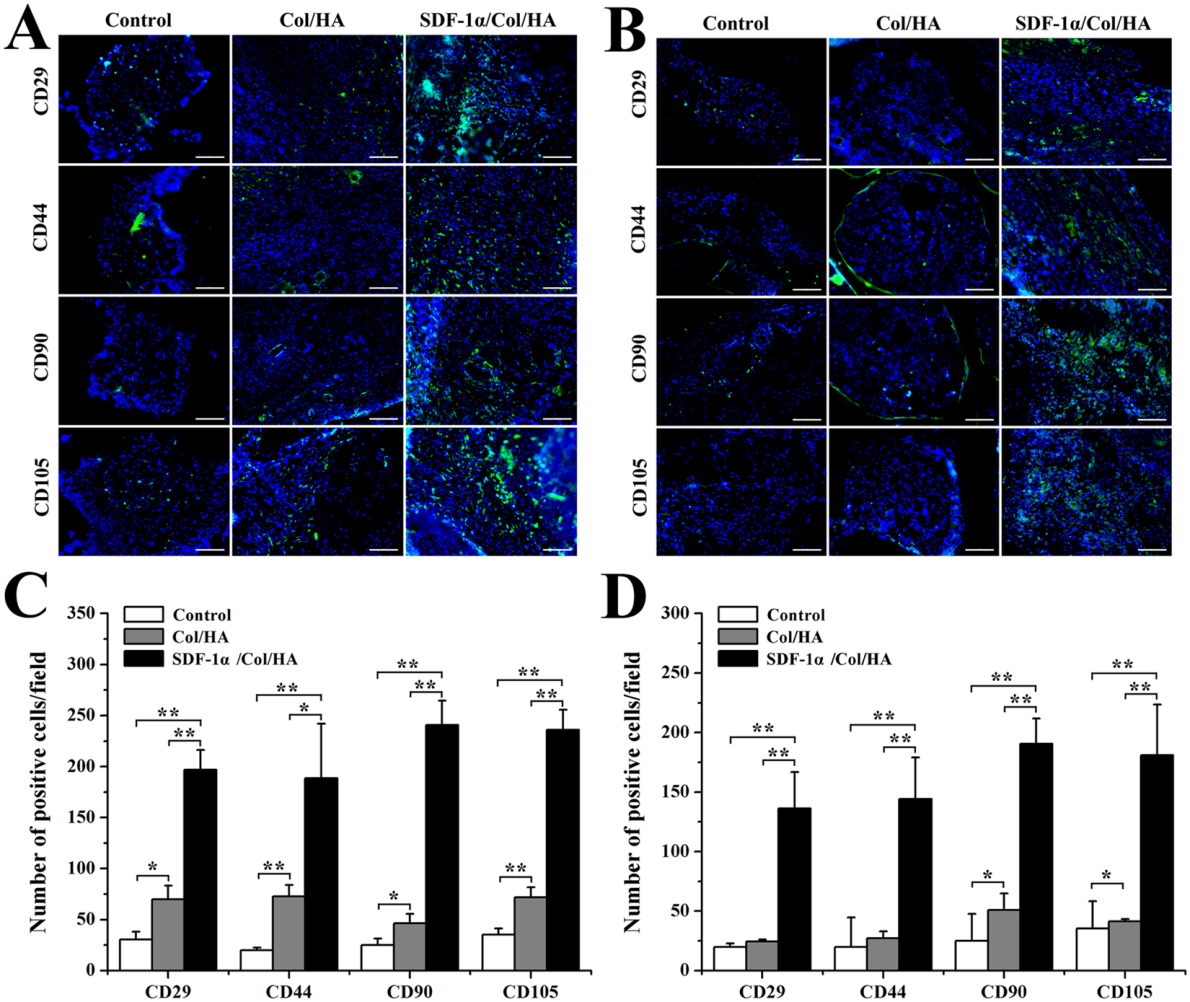
Utilization of stem cell markers to identify MSCs *in vivo* at day 5 and day 10. (**A**,**B**) Immunofluorescent staining of MSCs markers in the scaffolds after subcutaneous implantation at day 5 (**A**) and day 10 (**B**). The scale bar indicates 100 μm. (**C**,**D**) Quantitative data for cell number of CD29-, CD44-, CD90-, and CD105-positive cells on scaffolds after subcutaneous implantation at day 5 (**C**) and day 10 (**D**). *p < 0.05 and **p < 0.01, respectively.

**Figure 5:**
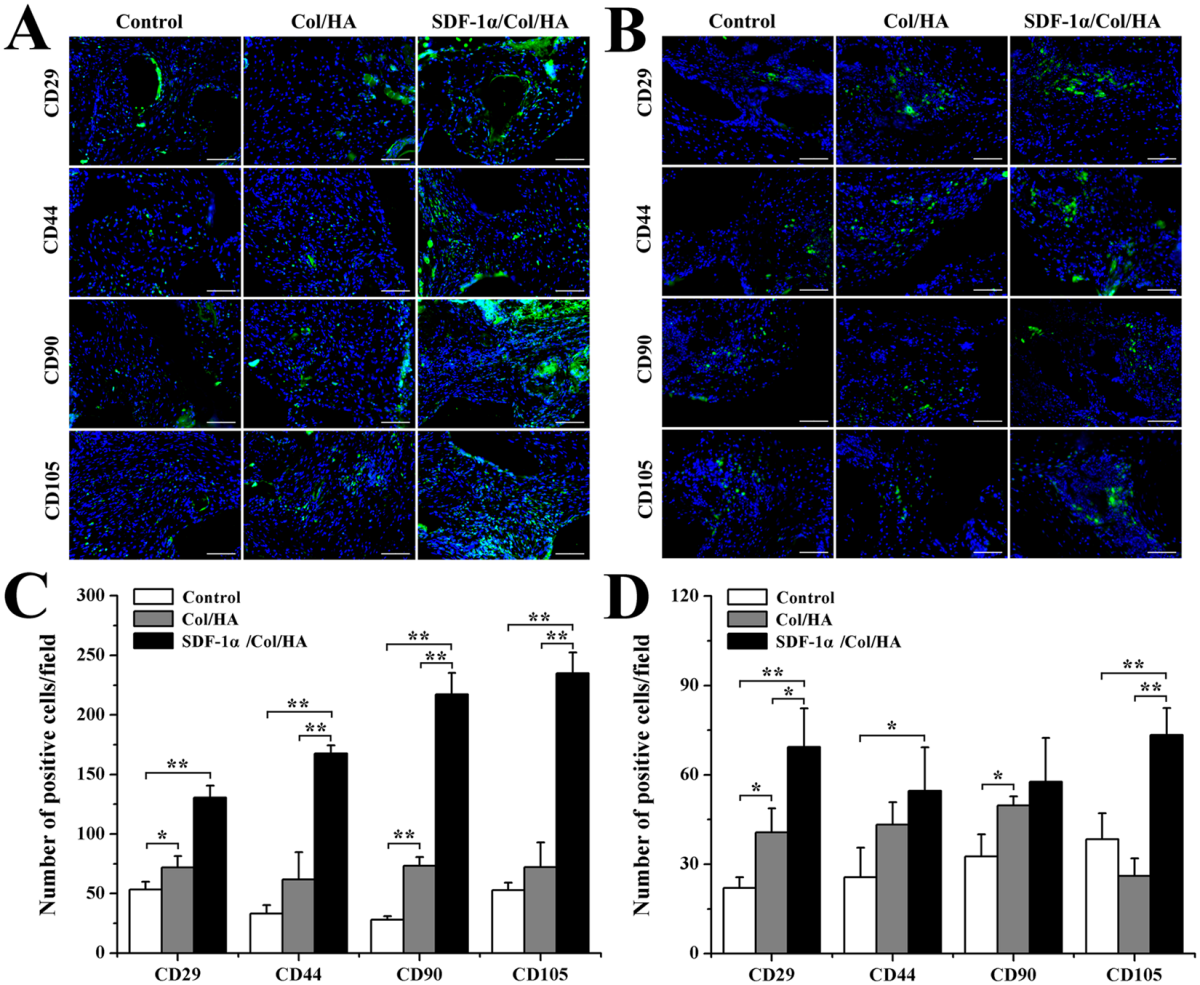
Utilization of stem cell markers to identify MSCs *in vivo* at week 4 and week 8. (**A**,**B**) Immunofluorescent staining of MSCs markers in the scaffolds after subcutaneous implantation at week 4 (**A**) and week 8 (**B**). The scale bar indicates 100 μm. (**C**,**D**) Quantitative data for cell number of CD29-, CD44-, CD90-, and CD105-positive cells on scaffolds after subcutaneous implantation at week 4 (**C**) and week 8 (**D**). *p < 0.05 and **p < 0.01, respectively.

The endogenous MSCs recruiting ability of SDF-1α/Col/HA scaffold was observed in the subcutaneous implantation area by testing the MSC markers. The positive staining of MSC markers CD29, CD44, CD90, and CD105 was found with different degrees in each group of scaffold at day 5, day 10, week 4, and week 8 after implantation in subcutaneous tissue of rats (Figs 4 and 5). At day 5, the control group and the Col/HA group showed few positive staining of MSC markers. On the one hand, the amount of positive cells in the Col/HA group was significantly more than that in the control group. On the other hand, the SDF-1α/Col/HA scaffold contained more positive cells than the control group and the Col/HA group (Fig. 4A,C). At day 10, the number of positive cells in SDF-1α/Col/HA scaffold had been enhanced significantly (Fig. 4B,D). At week 4, all groups showed an increasing tendency of positive cells and the SDF-1α/Col/HA group still had the highest number of positive cells compared with other groups (Fig. 5A,C). At week 8, all groups showed a decreasing tendency of positive cells, especially in the Col/HA group and SDF-1α/Col/HA group (Fig. 5B,D). Interestingly, the positive cells mainly clustered together in all the groups at 8 weeks. All these findings suggested that the SDF-1α/Col/HA scaffold can effectively improve the endogenous MSCs recruiting ability *in situ*. MSCs can sense the inflammation *in vivo* at the very onset^37^. After the scaffold was implanted into the subcutaneous tissue, the inflammatory response trigged a small amount of MSCs to all the scaffolds^15, 30^. By loading SDF-1α into a Col/HA scaffold, the number of MSCs was substantially increased in the SDF-1α/Col/HA group compared with other two groups at day 5 and day 10, and then decreased dramatically at 8 weeks. The decrease of the MSC density at 8 weeks is mainly due to decreased release of the SDF-1α from the SDF-1α/Col/HA at 8 weeks. The extent of MSC recruitment depended heavily on the duration of SDF-1α release profile from the SDF-1α/Col/HA scaffold (Fig. 1A), since the SDF-1α produced the magnification and extension of MSC recruitment. These results were in close agreement with those reported in the literature, although the exact origin of MSCs is still debated^18, 38, 39^. What is more, our recently study also found that high mobility group box-1 (HMGB-1) immobilized nanofibous scaffold can recruit stem cells through upregulation of SDF-1α *in vivo* ^40^.

Interestingly, compared with the control group, the Col/HA group also showed higher ability for MSC recruitment at four different time points. As shown in previous studies^41, 42^, collagen type I coating exhibited a dose-dependent chemotaxis of rabbit and human MSCs *in vitro* in the absence of soluble cytokines and the collagen fragments could mediate the migration of bone marrow stromal cells. The observed MSCs homing phenomenon in the Col/HA group may be related to the engagement of cellular integrin with binding sites on collagen matrix or the production of a homing agent by the collagen matrix itself^42^.

#### Evaluation of inflammatory cell infiltration

**Figure 6:**
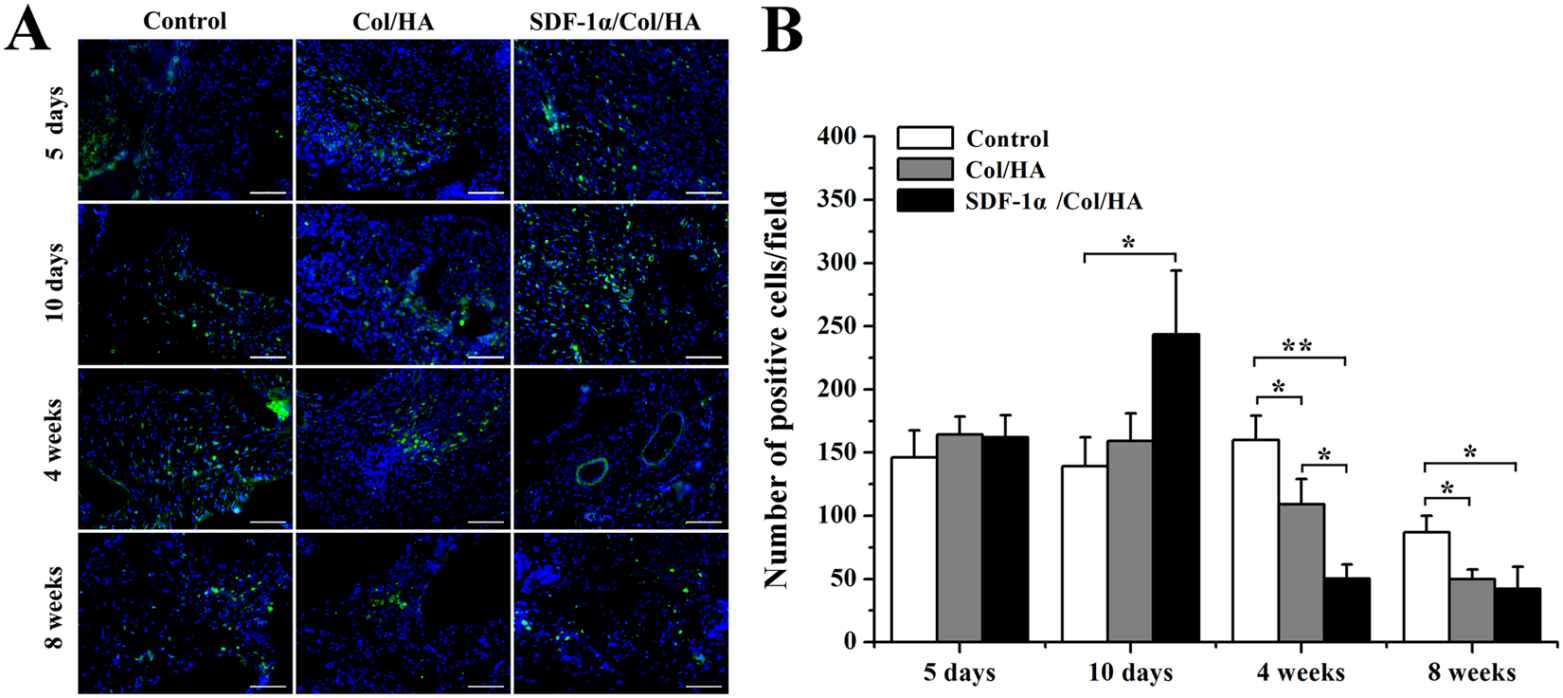
Immunofluorescent staining of CD68-positive macrophages (**A**) and quantitative data for cell number of CD68-positive macrophages (**B**) in the scaffolds after subcutaneous implantation for 5 days, 10 days, 4 weeks, and 8 weeks. The scale bar indicates 100 μm. *p < 0.05 and **p < 0.01, respectively.

Immunofluorescence staining of CD68-positive macrophages was performed to detect the infiltration of inflammatory cells in different scaffolds after implantation in subcutaneous tissue of rats for 5 days, 10 days, 4 weeks, and 8 weeks (Fig. 6). The amount of CD68-positive macrophages was significantly higher at day 10 in the SDF-1α/Col/HA group compared to the control group and Col/HA group (Fig. 6A). Significantly less CD68-positive cells were found in the SDF-1α/Col/HA group than other two groups at week 4 and week 8 (Fig. 6A). SDF-1α modified scaffold at week 4 resulted in significant increase in endogenous MSCs engraftment in the scaffold, which may contribute to the reduced accumulation of inflammatory cells in the scaffold at week 4 and week 8 (Figs 5 and 6). By quantifying the numbers of CD68-positive macrophages in the scaffolds after subcutaneous implantation at different time points, it is found that the SDF-1α/Col/HA scaffold elicited litter influence on inflammatory cell recruitment at day 5 (Fig. 6B). At day 10, the SDF-1α/Col/HA scaffold promoted the recruitment of inflammatory cells. However, compared with the control group and Col/HA group, SDF-1α release from the SDF-1α/Col/HA scaffold profoundly reduced inflammatory cell accumulation after subcutaneous implantation for 4 and 8 weeks. Our findings are consistent with the previous study, which showed that the SDF-1α can reduce the inflammatory responses *in vivo* ^43^.

#### Effect of SDF-1α/Col/HA scaffold on vascularization after subcutaneous implantation

**Figure 7:**
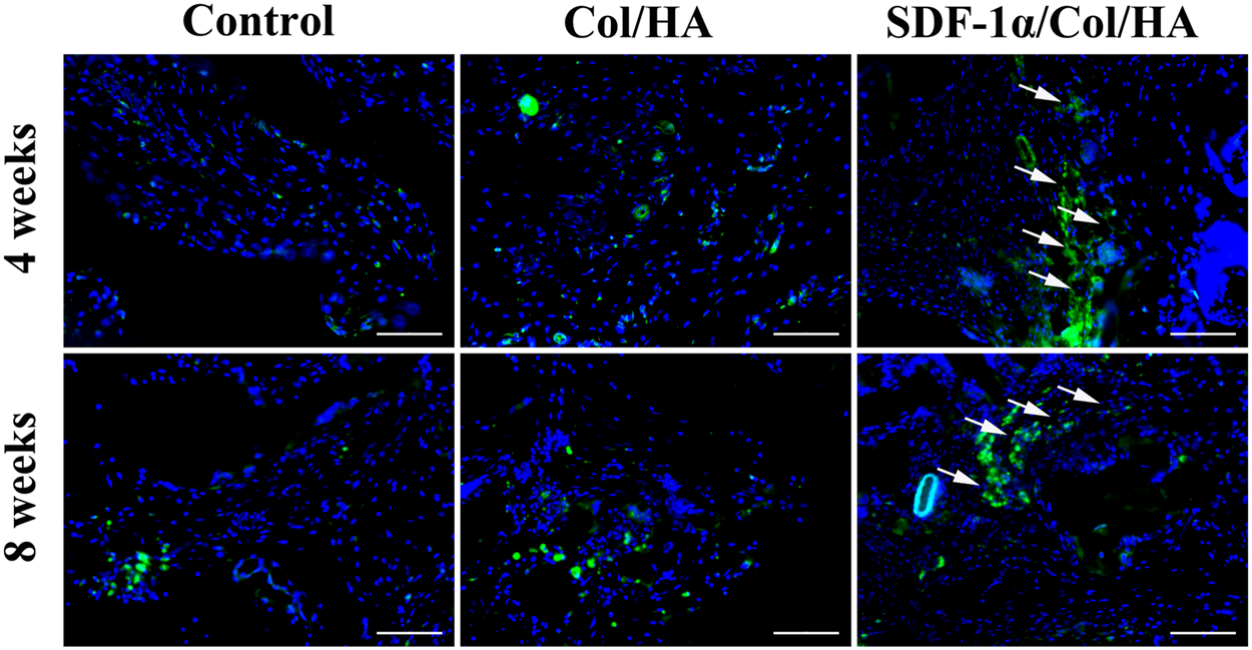
Immunofluorescent detection of CD34 in scaffolds after subcutaneous implantation. The white arrow indicates typical blood vessel. The scale bar indicates 100 μm.

In order to evaluate the effect of SDF-1α/Col/HA scaffold on vascularization, CD34 expression was detected by immunofluorescent staining after subcutaneous implantation for 4 and 8 weeks (Fig. 7). The number of CD34-positive cells in the SDF-1α/Col/HA scaffold was much higher than those in the decellularized bone scaffold and Col/HA scaffold. Meanwhile, the distribution of CD34-positive cells both in the Col/HA group and SDF-1α/Col/HA group showed varying degrees of aggregation. Compared with the control group (decellularized bone scaffold) and Col/HA group, typical blood vessels obviously appeared in the SDF-1α/Col/HA group. Our previous study^15^ proved that optimal matrix elastic modulus had a positive influence on the vascularization of the Col/HA scaffold. In addition to the effect of matrix mechanics on vascularization, there is evidence that SDF-1 can generate a angiogenic environment by upregulating of many cytokines related to angiogenic processes, including vascular endothelial growth factor (VEGF) and insulin-like growth factor binding protein 3 (IGFBP-3)^38, 44^ or directly recruits endothelial cells *in vivo* ^44^. Our data showed that the SDF-1α/Col/HA group had more CD34-positive cells than the Col/HA group, indicating that SDF-1α may magnify the vessel formation ability of the scaffold with optimal matrix elastic modulus. Besides, another important reason is that SDF-1α may involve in attracting of CD34-positive hematopoietic stem cells^23^ and endothelial progenitor cells^45^.

#### Effect of SDF-1α/Col/HA scaffold on osteogenesis after subcutaneous implantation

**Figure 8:**
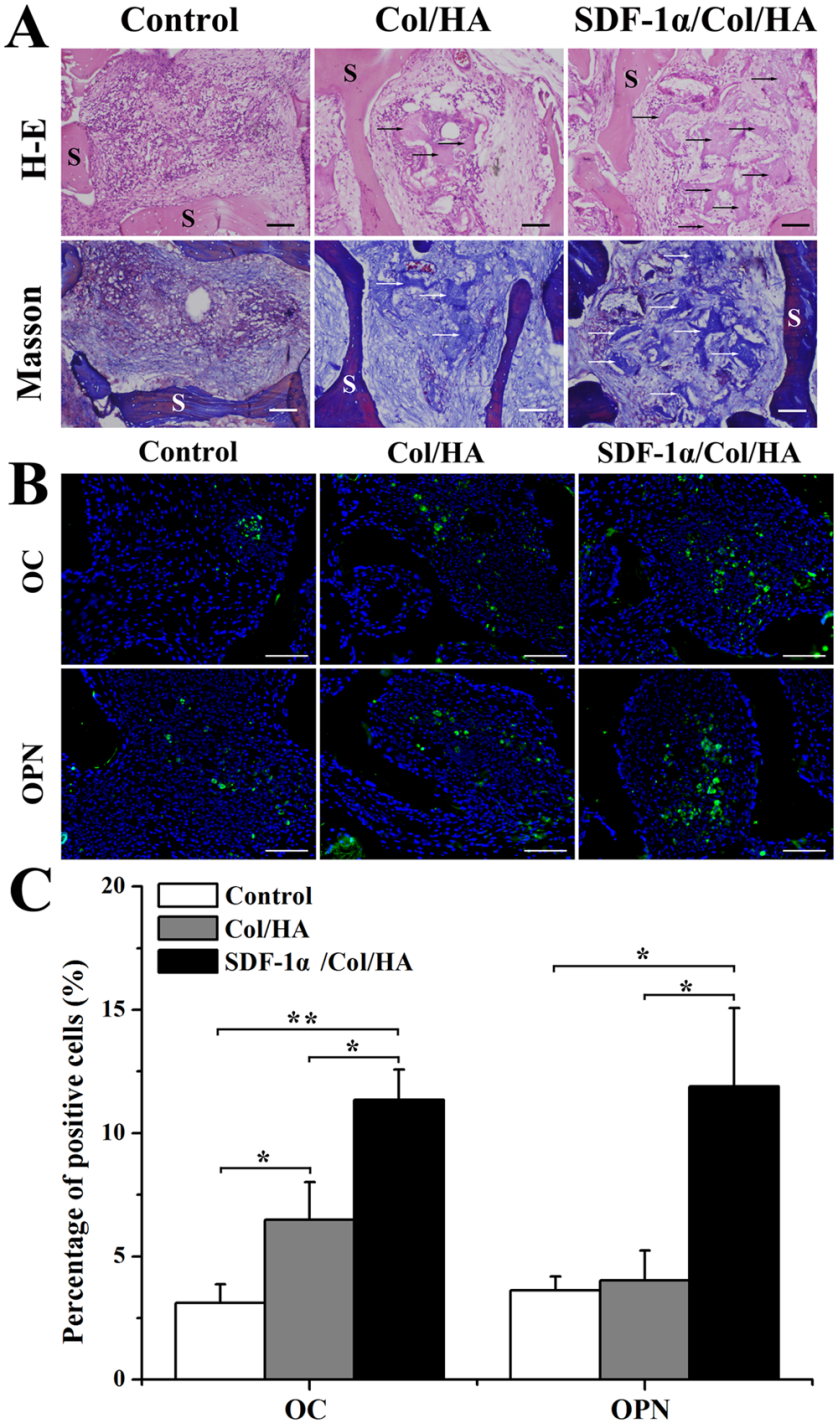
H-E staining and Masson trichrome staining (**A**) of constructs after subcutaneous implantation for 8 weeks. Arrows point to the area of new bone formation. S: scaffold. The scale bar indicates 50 μm. Immunofluorescent staining of OC and OPN in the scaffolds (**B**) and quantitative data for percentage of OC- and OPN-positive cells on scaffolds (**C**) after subcutaneous implantation for 8 weeks. The scale bar indicates 100 μm. *p < 0.05 and **p < 0.01, respectively.

To detect the cell infiltration, histological sections from all groups were examined by H-E staining after subcutaneous implantation for 8 weeks. As shown in Fig. 8A, the number of infiltrating cells in the control group was more than those in the Col/HA group and SDF-1α/Col/HA group. Masson trichrome staining and immunofluorescence staining of OPN and OC were performed to evaluate the osteogenic effect of the SDF-1α/Col/HA scaffold *in vivo* (Fig. 8). Masson trichrome staining showed that the control group had a large number of collagen fibrils (blue) and fibrous tissue (red). Compared to the control group, the collagen fibrils and fibrous tissue in the Col/HA group and SDF-1α/Col/HA group gradually decreased, and a small amount of new bone was produced. Meanwhile, the new bone area in the SDF-1α/Col/HA scaffold was significantly higher than those in the control group and Col/HA group (Fig. 8A). In addition, the percentage of positive immunofluorescence staining cells of OC in the Col/HA group and SDF-1α/Col/HA group was higher than that in the control group (Fig. 8B). OC expression in the SDF-1α/Col/HA group was also more evident compared with the Col/HA group. The percentage of positive immunofluorescence staining cells of OPN in the SDF-1α/Col/HA was the highest group among these three groups (Fig. 8B). The semi-quantitative of the percentage of OC- and OPN-positive cells in each field was further analyzed in Fig. 8C. Compared to the control group and Col/HA group, the percentage of OC-positive cells in the SDF-1α/Col/HA group had been increased 3.65-fold and 1.75-fold, respectively. Similarly, the SDF-1α/Col/HA group has a 3.28-fold and 2.96-fold increase in the percentage of OPN-positive cells compared to the control group and Col/HA group. Together, these data demonstrated that immobilization of SDF-1α onto the Col/HA scaffold can further enhance the osteogenic ability of the scaffold through recruiting more endogenous MSCs *in vivo*.

### Enhanced bone formation of SDF-1α/Col/HA scaffold in rabbit large bone defect model

**Figure 9:**
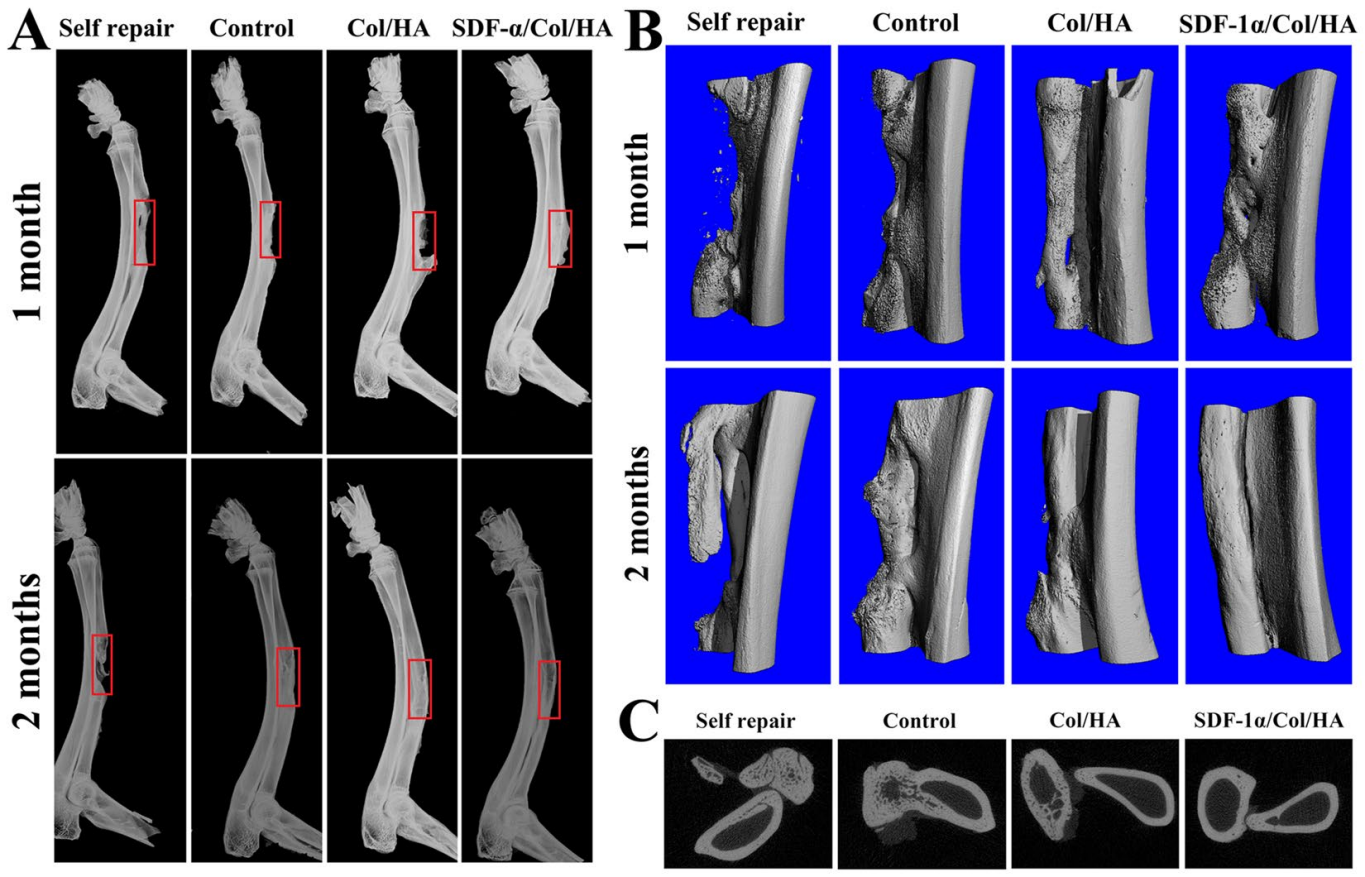
X-ray images (**A**) and μ-CT 3D images (**B**) of segmental radius at 1 month and 2 months with different implants. The red wireframe indicates the defective site. 2D section view images (**C**) of segmental radius at 2 months with different implants.

Figure 9A showed the qualitative assessment of X-ray radiographs to demonstrate the new bone formation in different groups at 1 month and 2 months. Little radiopacity appeared in the defect sites at 1 month after the operation in the self repair group (without any implant). However, obvious callus could be found in the distal ends of the defects in the control group (decellularized bone scaffold) and Col/HA group. Almost complete healing came out with the most extensive bone tissue formation throughout the entire defect in SDF-1α/Col/HA group at 1 month. At 2 months, the self repair group still could not achieve complete healing, but the radiopaque areas of binding part in the control group and Col/HA group were found gradually bigger and more consolidatory. Compared with the other three groups, the bone defect in the SDF-1α/Col/HA group appeared complete healing at 2 months (Fig. 9A).

The 3D restructured μ-CT images were conducted at 1 month and 2 months to evaluate the healing process of the long segmental defected bone in each group (Fig. 9B). Correspondingly, the self repair group showed the least bone binding and slowest healing rate without massive bone formation until 2 months. In the control group (decellularized bone scaffold), more neo-bone formation was present in the bone defects compared with the self repair group throughout the process. However, it seems that the defected bone has not been completely healed at 2 months. While both the Col/HA group and SDF-1α/Col/HA group exhibited comparatively good bridging and fast healing, in which the SDF-1α/Col/HA group performed slightly better than the Col/HA group with clearer boundary between ulna and repaired radius at 2 months (Fig. 9B). In addition, from the sectional view images in these four groups at 2 months, it can be seen that only the SDF-1α/Col/HA group achieved almost the same result as the normal bone (Fig. 9C). Our previous study^16^ has shown that the bone defects in the Col/HA group appeared complete healing at 3 months. Fortunately, only 2 months was needed to obtain the similar result in the SDF-1α/Col/HA group.

**Figure 10:**
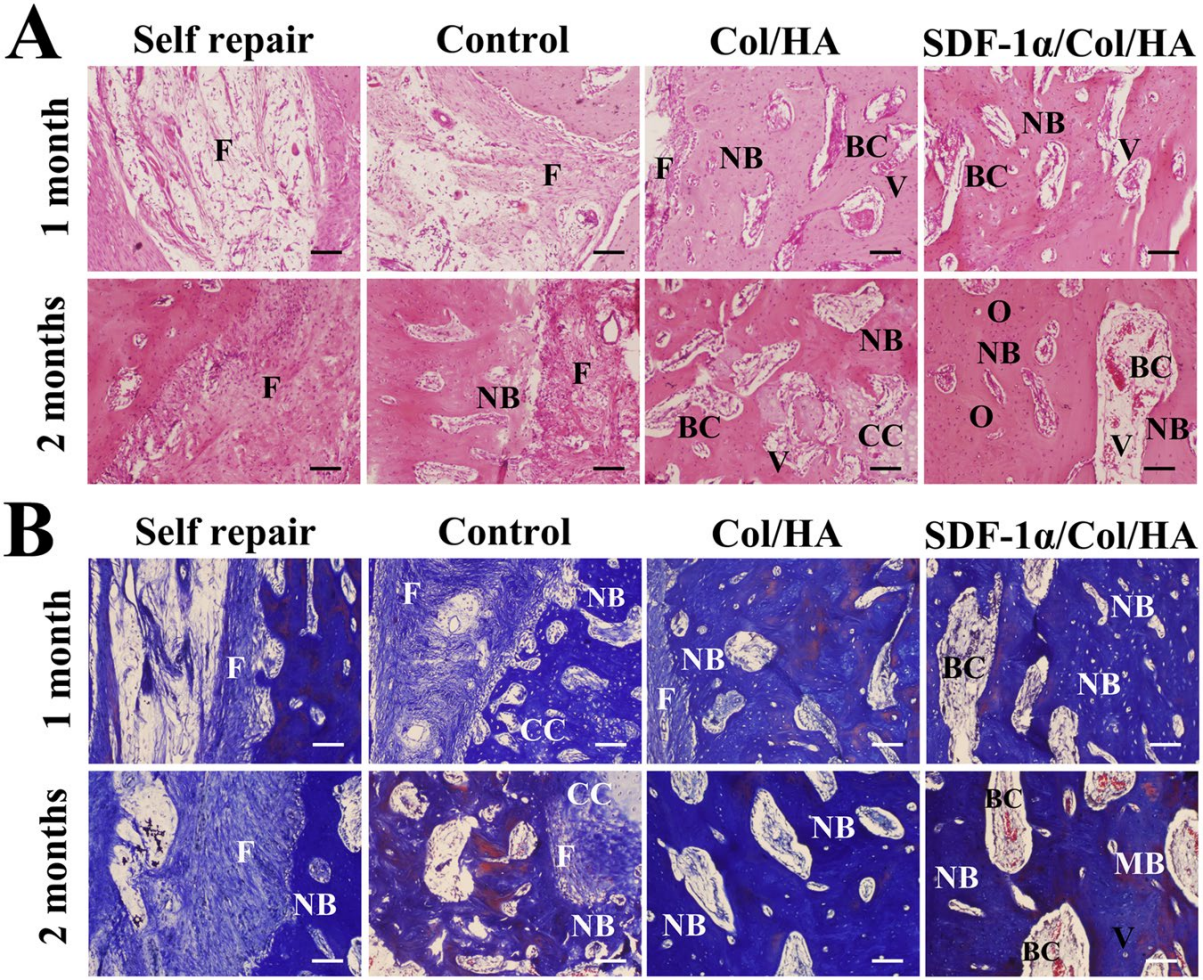
H-E staining (**A**) and Masson trichrome staining (**B**) of orthotopicly formed bone sections at 1 month and 2 months. F: fibrous tissue; NB: new bone; BC: blood cell; V: vessel; CC: chondrocyte; O: osteon; MB: mature bone. The scale bar indicates 50 μm.

The radiographic analysis and μ-CT analysis of the segmental defects were further supported by histological observation. H-E staining and Masson trichrome staining were performed to investigate the formation of new bone in the orthotopic segmental defect in each group at 1 month and 2 months. Figure 10 presented the photomicrographs of longitudinal sections (parallel to the long axis of bone) through the bone defect site in each group. The self repair group showed massive fibrous tissue in the defect till 2 months and did not form numerous mature new bones. In the control group (decellularized bone scaffold), segmental defect was filled with loose fibrous connective tissue at 1 month and a small amount of new bone formation was observed within loose fibrous connective tissue at 2 months. Compared to the above two groups, the Col/HA group and SDF-1α/Col/HA group had more pronounced new bone formation at 1 month and 2 months. Some blood cells were also found at some sites in the regenerated bone in these two groups. Meanwhile, the quantity of the new woven bone tissue in the SDF-1α/Col/HA group seemed to be greater than that in the Col/HA group at 1 month. At 2 months, the mature trabecular bone and the lamellar bone seemed more compact in the SDF-1α/Col/HA group which was also more evident by the formation of Haversian system, a typical osteon. These results suggested that the maximum bone regeneration level exhibited in the SDF-1α/Col/HA group was mainly due to the SDF-1α through mobilization and homing of endogenous MSCs to the bone defect site, and promoting bone regeneration *in situ*.

## Conclusion

Taken together, we successfully constructed and characterized a new SDF-1α/Col/HA scaffold with significant ability to recruit endogenous MSCs and promote osteogenic differentiation *in situ*. The *in vitro* experiments showed that SDF-1α/Col/HA scaffold can sustain adhesion and growth of rat MSCs and induce osteogenic differentiation. Subcutaneous implantation study revealed that the scaffold can recruit MSCs from subcutaneous tissue by SDF-1α release and promote them to differentiate into osteoblasts by matrix elasticity. More importantly, enhanced bone formation in rabbit large bone defect was achieved due to the efficient endogenous MSCs recruitment and osteogenic differentiation *in situ*. The SDF-1α/Col/HA scaffold has considerable potential as an engineered cell-free scaffold that can serve as a novel substitute in bone tissue engineering.

## Electronic supplementary material

Supplementary Information
