# Supplementary material for: Matrix elasticity-modified scaffold loaded with SDF-1α improves the in situ regeneration of segmental bone defect in rabbit radius
Source: Sci Rep. 2017 May 10;7:1672. doi: 10.1038/s41598-017-01938-3 (PMC5432001; doi:10.1038/s41598-017-01938-3)
Supplement: Supplementary file 1 — Supplementary Information [file 41598_2017_1938_MOESM1_ESM.pdf]

Matrix elasticity-modified scaffold loaded with SDF-1α improves the in situ regeneration of segmental bone defect in rabbit radius

Guobao Chen[1, 2] & Yonggang Lv[1, 2,*]

[1]Key Laboratory of Biorheological Science and Technology (Chongqing University), Ministry of Education, Bioengineering College, Chongqing University, Chongqing, 400044, P. R. China
[2]Mechanobiology and Regenerative Medicine Laboratory, Bioengineering College, Chongqing University, Chongqing, 400044, P. R. China
*Corresponding author, email: yglv@cqu.edu.cn

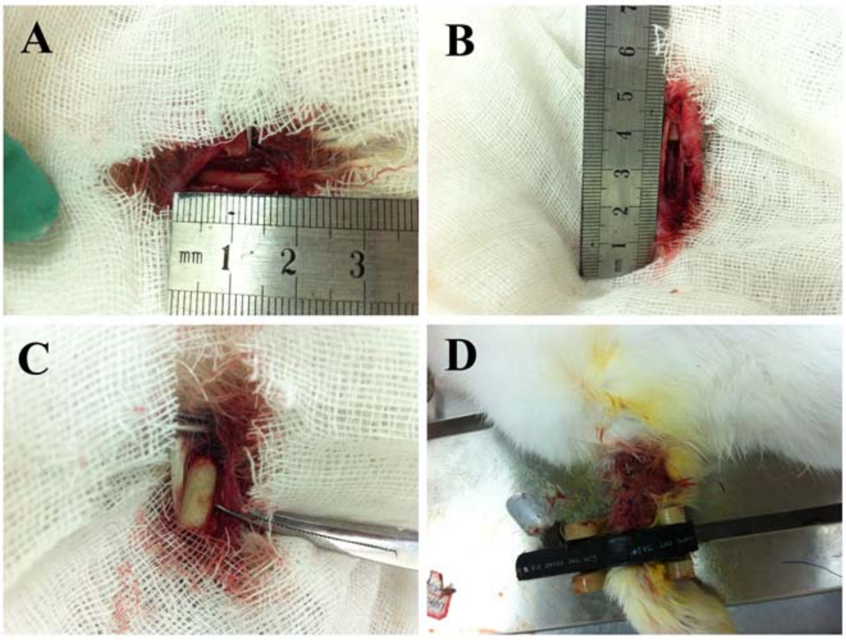

Supplementary Figure S1. Demonstration of the implantation surgery. (A) The radius of rabbit was exposed. (B) A defect was made in a rabbit radius. (C) A scaffold was inserted into the defect. (D) After implantation, the wound was sutured in full thickness.
